# A Qualitative Study on Ethics Education at Pharmacy Colleges in Japan Based on a Survey of Ethics Educators

**DOI:** 10.3390/pharmacy13020045

**Published:** 2025-03-18

**Authors:** Etsuko Arita, Yuko Masamura, Rieko Takehira

**Affiliations:** Laboratory of Medical Psychology Pharmaceutical Education Research Center, Kitasato University School of Pharmacy, Tokyo 108-8641, Japan; masamura.yuko@kitasato-u.ac.jp (Y.M.); takehirar@pharm.kitasato-u.ac.jp (R.T.)

**Keywords:** pharmacy education, Japan, qualitative research, ethics education

## Abstract

**Background**: In pharmacy education in Japan, efforts continue to develop a model for ethics education that fosters high ethical standards and the problem-solving skills essential for medical professionals. This study qualitatively analyzed the attitudes of ethics educators—those who teach ethics classes—to establish a model of ethics education for pharmacy colleges in Japan. **Methods**: This study analyzed open-ended responses from 32 universities to the question, “What do you think about ethics education provided by faculties of pharmaceutical sciences?” **Result**: The qualitative analysis revealed that ethics educators at pharmacy colleges in Japan believe in the potential of ethics education to nurture problem-solving skills and logical thinking. However, the educator’s question whether or not the students would be able to apply ethics content in clinical settings as medical professionals. Another issue is that faculties of pharmaceutical sciences lack the staff and expertise to teach ethics. In other words, the educators lack the wherewithal to break the logjam in ethics education through their efforts; hence, they are desperate for an ethics education model. **Conclusions**: Based on our findings, further research is needed to design strategies that can enhance the quality of pharmacy education in Japan.

## 1. Introduction

In clinical settings, medical professionals frequently encounter ethical problems with no clear right answer. Such problems require the ability to analyze the problem accurately and use logical thinking to derive an appropriate solution [1]. To that end, pharmacy schools should teach ethics to ensure that students, in addition to acquiring expert knowledge and skills, also develop the high ethical standards and logical thinking skills essential for medical professionals [2].

The World Federation for Medical Education states, in the 2015 edition of its Global Standards for Quality Improvement: Postgraduate Medical Education, that medical ethics should be part of the basic standards of medical education along with behavioral science, social medicine, and medical law when developing a curriculum [3]. The International Pharmaceutical Federation, which engages in pharmaceutical education from a global perspective, has established the ethical standards that pharmacists should uphold as medical professionals [4]. In the US, all pharmacy educators are required to have a general awareness of ethics. Indeed, training in ethical decision-making is a requirement for accreditation by the Accreditation Council for Pharmacy Education and the American Society of Health-System Pharmacists [5,6].

Pharmacy education in Japan, however, has a long history rooted in providing a strong foundation in chemistry, which is essential for training researchers in fundamental scientific fields. In March 2007, Japan began transitioning to a system of undergraduate faculties aimed at training medical professionals by introducing six-year programs in pharmaceutical science. Japan’s Model Core Curriculum for Pharmacy Education emphasizes the importance of developing “an ethical mindset conducive to respecting and safeguarding the rights of patients and the consumers” as a key competency for pharmacists [7]. However, there is an ongoing search for an effective model of ethics education in pharmaceutical sciences that can prepare students to meet high ethical standards and acquire the problem-solving skills necessary for their careers as medical professionals [8,9,10].

To understand the present state of ethics education in pharmacy colleges in Japan and to establish a framework for ethics education that can be implemented in pharmacy schools, we conducted a nationwide survey on the state of ethics education, including aspects such as the year of study in which it is taught, the instructors responsible, the content covered, and the assessment methods used. The findings have already been presented at the 143rd Annual Meeting of the Pharmaceutical Society of Japan [11,12].

This study qualitatively analyzed the attitudes of ethics educators—those who teach ethics classes. This study is the first to focus on pharmacy educators in Japan.

## 2. Materials and Methods

### 2.1. Survey Method and Sample

Between 8 March 8 and 22 March 2021, an online questionnaire survey was conducted among ethics educators at 77 pharmacy colleges in Japan. A qualitative analysis was conducted on the open-ended responses to the questionnaire item, “What do you think about ethics education provided by faculties of pharmaceutical sciences?”.

### 2.2. Data Analysis

The Steps for Coding and Theorization were used in the qualitative analysis of the text data [13,14,15]. This involves the following four-step process for analyzing text data: First, the text is segmented. Second, the segments are encoded by (1) identifying keywords/phrases; (2) replacing the keywords/phrases with words/phrases not found in the text data; (3) replacing the alternative words/phrases with descriptive concepts; and (4) identifying themes and constructs from the concepts. Third, a storyline is written connecting the themes and constructs. Fourth, a theory is developed based on the storyline [16,17,18]. In theoretical descriptions, themes and construct expressions are modified while maintaining their original meaning to ensure coherence and fluency in writing.

In this study, the following process was adopted:

1. Data input: The text data (the questionnaire responses) were segmented and arranged into a table;
2. Encoding: The text was replaced with alternative words/phrases (codes) according to the context;
3. Categorization: Similar codes were grouped into categories;
4. Storyline: For each category, a storyline was written incorporating every code in the category;
5. Theoretical description: A theoretical description was crafted to encapsulate all the storylines;

Regarding data input, when a respondent’s descriptive response included multiple semantic units, a separate segment was created for each unit. For encoding, the four-step encoding process was followed to identify the concepts. After crafting a theoretical description, each category was given a label describing the category’s content, collectively representing attitudes toward ethics education as provided by the faculties of pharmaceutical sciences.

The initial analysis was performed by E.A., R.T., and Y.M.; two of the co-authors had extensive experience in qualitative research. E.A. organized the text data into a table, which was checked by R.T. and Y.M. E.A. and R.T. categorized the codes, which were checked by Y.M. E.A. and Y.M. developed the storyline, which was checked by R.T. Subsequently, the validity of the analysis was evaluated. When the authors differed in opinion, they discussed and re-examined the content in question. Once they reached a consensus, the study proceeded to the next step of the analysis, ensuring validity.

E.A., R.T., and Y.M. work at the same institution and, therefore, engaged in regular face-to-face discussions to critically review and refine this research.

### 2.3. Ethical Considerations

The study required no ethical screening in Japan, as it did not fall under the Ethical Guidelines for Medical and Biological Research Involving Human Subjects [19]. Participation in the online survey (conducted among pharmacy schools in Japan) was voluntary, and no personal information was collected. The colleges sampled received a written briefing stating the results would remain anonymous. Participation in the survey was interpreted as an indication of consent to engage in the study.

## 3. Results

The open-ended responses from 32 colleges out of the 77 surveyed with the question “What do you think about ethics education provided by faculties of pharmaceutical sciences?” were analyzed. The effective response rate was 41.5%.

We have identified six perspectives and fifty-five concepts based on the participants’ responses. Perspectives and concepts are denoted by square ([]) and angle brackets (<>).

Table 1 presents the representative perspectives, concepts, and theoretical descriptions identified by the qualitative analysis.

[An ethical education system that incorporates diversity and focuses on society] included five concepts: <students’ ethical interpretations tend to be self-centered>; <ethical standards form the basis of careers as medical professionals, as they are responsible members of society>; <a lack of socially aware ethics education>; <a desire for ethics education that is responsive to societal changes>; and <a need for an educational system that incorporates diversity>.

[Instill the basic ethical awareness necessary for a medical professional or researcher] included four concepts: <opportunities to foster the ethical standards that form the basis of a career as a medical professional or researcher>; <the importance of balancing medical science and research when inculcating ethical standards>; <the ethics curriculum is ill-suited to the needs of students who do not aspire to pursue a medical career>; and <the difficulty of maintaining student motivation>.

[The pharmacy education community has no shared objectives] included eight concepts: <understanding the importance of teaching medical ethics necessary for pharmacy educators>; <university faculty staff are uninterested in changes in pharmacy education>; <views about ethics education differ between different fields>; <a poor understanding of what ethics education is required in pharmacy education>; <the prevailing educational environment impedes integrated ethics education>; <pharmacy educators tend to believe that it is unnecessary to train ethics specialists>; <the pharmacy education community has no shared objectives for ethics education>; and <pharmacy ethics education is far from ideal>.

[Concerns about whether or not these teachings are applicable in clinical practice] included 17 concepts: <ethics education should foster professionalism in medical professionals>; <fostering ethical sensitivity should be an educational objective>; <a desire to instill ethical standards that all medical professionals should uphold>; <as a faculty for training medical professionals, the faculty should give priority to ethics education>; <a lack of awareness as a faculty for training medical professionals>; <a discrepancy in understanding between pharmacy educators>; <ethics education is not integrated with the training of medical professionals>; <the ethics education that pharmacists require>; <it is necessary to understand that ethics education is for the patient>; <it is necessary to disseminate patient-oriented ethical standards>; <ethics education is a necessary preliminary step in clinical practice>; <it would be effective to incorporate the required educational content into practical training>; <ethics education should be relevant to clinical practice>; <there is a lack of mutual respect between active pharmacists and university faculty staff>; <there is a disconnect between ethics education and clinical practice>; <there are too few opportunities to encounter the diverse values that one would encounter through communication>; and <there are concerns about whether or not these teachings are applicable in clinical practice>.

## 4. Discussion

We asked ethics educators at pharmacy schools in Japan to tell us what they think about the ethics education provided by faculties of pharmaceutical sciences and qualitatively analyzed their responses.

The ethics educators believed that ethics education, as part of a pharmaceutical science course, should constitute an ethical education system that incorporates diversity and focuses on society and should instill the basic ethical awareness necessary for a medical professional or researcher.

The first perspective emphasizes that ethics educators should equip their students with the basic ethical standards that responsible members of society should uphold before introducing them to specialized knowledge and skills. This further implies that educators should encourage faculty staff to be aware of societal changes. The existing literature highlights the importance of introducing a sociocultural dimension at the undergraduate level. Since pharmacists will engage with patients and members of the public of different ages, conditions, and values; they should have the cultural competency to accommodate such diverse needs and values [20,21,22].

From the second perspective, we identified the following concept describing ethics education at faculties of medical science: <the importance of balancing medical science and research when inculcating ethical standards>. To relate this to the literature, medical professionals (pharmacists) require research skills to transform clinical inquiries encountered in clinical settings into research questions, generating scientific evidence [23]. However, since the objectives of medical practice and research differ, the ethics of a medical professional can conflict with the ethics of a researcher [24,25,26]. If medical professionals are to engage in research appropriately, with an awareness of the differences in objectives between medical practice and research, they need to be taught at college about a wide range of ethical matters [27,28].

However, in Japanese pharmacy schools, the pharmacy education community has no shared objectives for ethics education, a situation that makes ethics educators feel concerned about whether or not these teachings are applicable in clinical practice.

The belief that the pharmacy education community has no shared objectives for ethics education shows that pharmaceutical science educators lack a common understanding of the elements of ethics education that are necessary for training pharmacists. Moreover, despite the significant sociocultural changes relevant to pharmacists and pharmaceutical science education, the results suggest that university faculty staff are uninterested in understanding what models of ethics education are necessary to meet societal needs. The results also suggest that ethics educators face a dilemma in which they understand the importance of ethics education in faculties of pharmaceutical sciences but feel unsupported by the university. Okamoto reported that medical schools in the US incorporate medical ethics into their medical training programs [29]. Japanese faculties of pharmaceutical sciences should follow this approach; they should develop a comprehensive model of ethics education founded upon the shared objective of fostering high-quality medical professionals.

The concerns about whether or not the teaching is applicable in clinical practice indicate that ethics educators fully acknowledge the importance of ethics education in the fostering of the professionalism necessary for medical professionals but also prioritize learning ethics through practical training following graduation, whereby the learner learns to deal with the ethical issues encountered in clinical settings. In Japan, however, because it remains poorly understood that faculties of pharmaceutical sciences are responsible for the education and training of healthcare professionals, a gap in awareness about the ethics education necessary for pharmaceutical science students exists among university faculty staff and between staff and pharmacists. This situation may explain why ethics educators believe that ethics teaching at faculties of pharmaceutical sciences has limited use in clinical practice. Whether or not the person can spot an ethical issue depends on their ethical sensitivity, which is correlated with the sense of responsibility as a medical professional [30,31]. In a study on ethical sensitivity, Aoyagi concluded that medical professionals’ ethical sensitivity is a “synthetic capacity,” which includes “not only explaining ethical problems but also taking a positive stance to face up to these problems” [32]. Other studies argue that training in ethical sensitivity helps foster a pharmacist’s professionalism [33]. Other studies note that pharmacists typically have few opportunities to learn about ethics when they were students. Hence, there is an urgent need to train active pharmacists to achieve consensus on the ethics education to be provided at faculties of pharmaceutical sciences [34,35].

Despite this situation, ethics educators remain optimistic, believing in the potential of ethics education to cultivate logical thinking skills. To break through the impasse, ethics educators want an advocate to present a teaching model that can resolve the current challenges.

The perspective of believing in the potential of ethics education to cultivate logical thinking skills implies that ethics educators have reservations about the conventional knowledge-driven curriculum. It also implies that they believe in the potential of ethics education to foster logical thinking and problem-solving skills, thereby addressing the problematic legacy of the conventional knowledge-driven approach. Since the ethical problems encountered by pharmacists in clinical settings have no clear right answer, pharmacists need to communicate and confer with the parties concerned with finding the best possible solution for the patient in question, which requires logical thinking and problem-solving skills. However, as pharmacy college students in Japan need to acquire a national pharmacist license, the education offered in faculties of pharmaceutical sciences tends to rely on knowledge-driven teaching, setting tasks with right and wrong answers [36]. The literature suggests that students’ problem-solving skills should be honed through active learning, including group discussions about specific cases [37,38,39]. Thus, logical thinking should be taught at the undergraduate level.

As for [wanting an advocate to present a teaching model], this implies that ethics educators agree with the principle that active learning is essential in linking knowledge with practice but feel that such an approach is unfeasible in practice owing to time constraints, a lack of manpower, and insufficient teaching ability. Ethics educators may also feel daunted by how to assess students’ performance in ethics tasks when this performance cannot be quantified. This problem also applies to other areas of medical education, and many studies have examined interventions for helping learners overcome it [40,41,42,43]. However, faculties of pharmaceutical science lack the staff and expertise for teaching ethics given their short history in pharmacy education. This situation means that ethics educators lack the wherewithal to break the logjam in ethics education through their efforts. Hence, there is a need for outside assistance and for academic societies and other education leaders to present a model of ethics education.

This study indicates that while ethics education is crucial, there are several barriers to its integration into pharmacy programs in Japan. A significant barrier is the gap in understanding between pharmacy faculty members and pharmacists in clinical settings. To address this gap, creating opportunities for dialog among faculty members and fostering collaboration with local healthcare institutions is essential. Surveys targeting educators, such as the one conducted in this study, are valuable for enhancing the awareness of those involved in ethics education.

This study has certain limitations. First, we asked ethics educators at pharmacy colleges across Japan to freely describe their attitudes. This question format might have led to a situation where faculty members with little interest in the topic may have refrained from responding. Conversely, this format helped to elicit more genuine opinions about ethics education. Second, in this analysis, we did not consider differences in responses due to respondents’ attributes or university curricula.

## 5. Conclusions

This study provides valuable insights into the perspectives of ethics educators at pharmacy colleges in Japan regarding the current state of ethics education in pharmacy, highlighting key issues and potential areas for improvement. These educators believe in the potential of ethics education to foster problem-solving skills and logical thinking. However, beliefs about ethics education differ among university faculty staff and between universities and clinical settings, and educators question whether or not students will be able to apply the ethics content in clinical settings as medical professionals. Addressing this requires fostering dialog and collaboration with healthcare institutions. Surveys targeting educators help raise awareness of ethics education.

Further research is necessary to derive recommendations for improving pharmacy ethics education in Japan, with a focus on cultivating pharmacists who meet high ethical standards and have the problem-solving skills necessary for a medical professional.

## Figures and Tables

**Table 1:** The representative perspectives, concepts, and theoretical descriptions.

Perspective	Concept	Theoretical Description
**[An ethical education system that incorporates diversity and focuses on society]**	<students’ ethical interpretations tend to be self-centered>, <ethical standards form the basis of careers as medical professionals, as they are responsible members of society >, <a lack of socially aware ethics education>, <a desire for ethics education that is responsive to societal changes>, <a need for an educational system that incorporates diversity>.	・ Regarding students’ tendency toward adopting self-centered ethical interpretations, the educators believe that, as responsible members of society, students should be equipped with ethical standards, which form the basis of a career as a medical professional. ・ There is a lack of socially aware ethics education, and a model of ethics education that is responsive to societal change is desired. ・ Responsiveness to societal change requires an educational system that incorporates diversity.
**[Instill the basic ethical awareness necessary for a medical professional or researcher]**	<opportunities to foster the ethical standards that form the basis of a career as a medical professional or researcher>, <the importance of balancing medical science and research when inculcating ethical standards>, <the ethics curriculum is ill-suited to the needs of students who do not aspire to pursue a medical career>, <the difficulty of maintaining student motivation>.	・ Ethics education at faculties of pharmaceutical sciences should serve as an opportunity to foster the ethical standards that form the basis of a career as a medical professional and researcher, and it is important to balance medical science and research when inculcating ethical standards. ・ It is difficult to maintain students’ motivation when the ethics curriculum is ill-suited to the needs of students who do not aspire to pursue a medical career.
**[The pharmacy education community has no shared objectives]**	<understanding the importance of teaching medical ethics necessary for pharmacy educators>, <university faculty staff are uninterested in changes in pharmacy education>, <views about ethics education differ between different fields>, <a poor understanding of the ethics education required in pharmacy education>, <the prevailing educational environment impedes integrated ethics education>, <pharmacy educators tend to believe that it is unnecessary to train ethics specialists>, <the pharmacy education community has no shared objectives for ethics education>, <pharmacy ethics education is far from ideal>.	・ The importance of medical ethics education needs to be understood; however, some faculty staff are uninterested in the changes in pharmacy education and views about ethics education differ among different fields. ・ Pharmacy educators tend to believe that it is unnecessary to train ethics specialists due to a poor understanding of what ethics education is required in pharmacy education coupled with a prevailing educational environment that impedes integrated ethics education. ・ As the pharmacy education community has no shared objectives for ethics education, pharmacy ethics education is far from ideal.
**[Concerns about whether or not these teachings are applicable in clinical practice]**	<ethics education should foster professionalism in medical professionals>, <fostering ethical sensitivity should be an educational objective>, <a desire to instill ethical standards that all medical professionals should uphold>, <as a faculty that trains medical professionals, the faculty should give priority to ethics education>, <a lack of awareness as a faculty for trains medical professionals>, <a discrepancy in understanding between pharmacy educators>, <ethics education is not integrated with the training of medical professionals>, <the ethics education that pharmacists require>, <it is necessary to understand that ethics education is for the patient>, <it is necessary to disseminate patient-oriented ethical standards>, <ethics education is a necessary preliminary step in clinical practice>, <it would be effective to incorporate the required educational content into practical training>, <ethics education should be relevant to clinical practice>, <there is a lack of mutual respect between active pharmacists and university faculty staff>, <there is a disconnect between ethics education and clinical practice>, <there are too few opportunities to encounter the diverse values that one would encounter through communication>, <there are concerns about whether or not these teachings are applicable in clinical practice>.	・ Faculties of pharmaceutical sciences are faculties that train medical professionals, and as such, they should give priority to ethics education. By doing so, they can instill the ethical standards that all medical professionals should uphold, with the educational objective of fostering the professionalism and ethical sensitivity of medical professionals. ・ Some teaching staff at faculties of pharmaceutical sciences fail to recognize that such faculties are intended for training medical professionals. Consequently, the discrepancy in understanding among pharmacy educators means that ethics education is not incorporated into the training of medical professionals. ・ To develop the ethics education that pharmacists require, it is necessary to disseminate the perspective that ethics education is for the patient and patient-oriented ethical standards. ・ Ethics education relevant to clinical practice should be delivered with an effective teaching model that integrates the required educational content with practical training as a preliminary step to clinical practice. ・ However, with a lack of mutual respect between active pharmacists and university faculty staff, there is a disconnect between ethics education and clinical practice. ・ Moreover, with the lack of opportunities to encounter the diverse values that one would encounter through communication in clinical settings, educators have concerns about whether or not pharmacy teaching would have practical value in clinical settings.
**[Believing in the potential of ethics education to cultivate logical thinking skills]**	<the problems of knowledge-centered exams>, <misgivings about education based on the assumption of definite right answers>, <the education content should foster logical thinking skills>, <ethics education is becoming increasingly important as a way of developing problem-solving skills>, <ethics education as a course subject that is integrated and independent>, <delayed because obtaining licenses is prioritized>, <belief in the potential of ethics education to help address the insufficiency of thinking skills and problem-solving skills caused by a knowledge-driven curriculum>	・ In the face of the problems of knowledge-centered exams and misgivings about education based on the assumption of definite right answers, pharmacy educators believe that the education content should foster logical thinking skills. ・ Ethics education is becoming increasingly important as it fosters problem-solving skills, especially given the expectation that it can help address the deficiency in critical thinking and problem-solving skills caused by a knowledge-driven curriculum. ・ To that end, ethics as a course subject needs to be integrated, independent, and part of a vertically and horizontally structured education system; however, this is delayed because obtaining licenses is prioritized.
**[Want an advocate to present a teaching model]**	<active learning is necessary>, <active learning is unfeasible in practice due to time constraints and lack of manpower>, <educators would struggle to use an active-learning approach because of a lack of experience>, <need for practical examinations that are broad enough to encompass ethics>, <performance assessments would be effective>, <non-standardized curriculum content and assessment>, <difficulties in objectively assessing students’ ethical standards>, <insufficient teaching ability>, <educators need to engage in self-improvement activities>, <need for opportunities to learn teaching and assessment approaches>, <shortage of ethics educators in pharmacy education>, <the right educator is unavailable, resulting in a logjam>, <want someone to present a model for pharmacy ethics education>, <hope that someone will take on an advocacy role in pharmacy education>	・ Although pharmacy educators recognize the need to adopt an active-learning approach in ethics education, they feel that such an approach is unfeasible in practice owing to time constraints, lack of manpower, and insufficient teaching ability. ・ Assessment in ethics education should involve practical examinations that are broad enough to encompass ethics, and performance assessments would be effective. ・ The non-standardized curriculum content and assessment method poses challenges concerning objectively assessing students’ ethical standards. ・ Given that educators should engage in self-improvement to address the insufficient teaching ability, opportunities to learn teaching and assessment approaches should be available. ・ The shortage of ethics educators in pharmacy education means that the right educator is unavailable, resulting in a logjam. ・ The educators want someone to present a model for pharmacy ethics education and pin their hopes on someone taking on an advocacy role.

## Data Availability

No new data were generated during this study.
